# Body dysmorphic disorder in adolescents: nosology, evolution and treatment. A case report

**DOI:** 10.1192/j.eurpsy.2025.1159

**Published:** 2025-08-26

**Authors:** L. Mallol, P. Del Sol, L. García-Murillo

**Affiliations:** 1 Hospital Puerta de Hierro, Majadahonda, Spain

## Abstract

**Introduction:**

Body dysmorphic disorder is a diagnostic entity within the somatoform disorders of the obsessive-compulsive spectrum according to the DSM-V. Its prevalence is between 0.7% and 2.5%, being more frequent in men and usually beginning in adolescence or early adulthood. Its central symptom is cognitive distortions regarding appearance, with concern about imaginary physical defects, slight or even invisible to others and in some cases close to a delusional condition.

**Objectives:**

To delve deeper into the clinical aspects and nosological conditions through the description of a clinical case.

**Methods:**

This is a 16-year-old patient who was admitted three times to the Acute Psychiatric Unit of the Puerta de Hierro Hospital due to behavioral disorders in the context of dysmorphophobia with intense concern about the shape of his nose. The patient gradually abandoned his daily routines, stopped attending classes at school and isolated himself from his social circle.

He is the youngest of two sisters. He lives with his parents and sister. He had good academic results until the time of diagnosis.

The patient’s concern about his nose began at about age 15, coinciding with the diagnosis of leukemia of his mother, who was undergoing chemotherapy treatment. The patient reports that the symptoms have increased until causing significant functional limitation.

He says that his nose is very large and he doesn’t allow anyone to see it. He leaves the house wearing a mask, performs rituals of checking it for hours in front of the mirror and has even manipulated his nose with objects to try to deform it. He appears to have a delusional component with significant emotional and behavioural repercussions.

**Results:**

Several pharmacological treatments were started, including SSRIs (sertraline) and various antipsychotics (risperidone, pimozide and olanzapine), with little response in all cases. Psychotherapeutic treatment was carried out in a day hospital for adolescents, but this was also unsuccessful. Finally, he was involuntarily transferred to a Medium Stay Unit given the significant impact of the symptoms on his life, showing progressive improvement after several months of treatment.

**Conclusions:**

The nosology of TDC has evolved: the DSM IV specified a delusional variant within the psychotic spectrum and a non-delusional one within the somatoform spectrum. The DSM V integrates it within the obsessive-compulsive spectrum. In order to assess a correct treatment, a continuum could be established between TDC and eating disorders. Both present similar symptoms: dissatisfaction and distortion with body image, need for continuous checking and concern about imperfections in perceived appearance. Given the chronicity of TDC and the tendency to abandon treatment in the first months, long-term follow-up by a multidisciplinary team is necessary, as well as psychoeducation and the establishment of a solid therapeutic alliance.

**Disclosure of Interest:**

None Declared

